# Identification and Evaluation of Reference Genes for Accurate Transcription Normalization in Safflower under Different Experimental Conditions

**DOI:** 10.1371/journal.pone.0140218

**Published:** 2015-10-12

**Authors:** Dandan Li, Bo Hu, Qing Wang, Hongchang Liu, Feng Pan, Wei Wu

**Affiliations:** Agronomy College, Sichuan Agricultural University, Wenjiang 611130, Chengdu, Sichuan, P. R. China; University of Heidelberg, GERMANY

## Abstract

Safflower (*Carthamus tinctorius* L.) has received a significant amount of attention as a medicinal plant and oilseed crop. Gene expression studies provide a theoretical molecular biology foundation for improving new traits and developing new cultivars. Real-time quantitative PCR (RT-qPCR) has become a crucial approach for gene expression analysis. In addition, appropriate reference genes (RGs) are essential for accurate and rapid relative quantification analysis of gene expression. In this study, fifteen candidate RGs involved in multiple metabolic pathways of plants were finally selected and validated under different experimental treatments, at different seed development stages and in different cultivars and tissues for real-time PCR experiments. These genes were *ABCS*, *60SRPL10*, *RANBP1*, *UBCL*, *MFC*, *UBCE2*, *EIF5A*, *COA*, *EF1-β*, *EF1*, *GAPDH*, *ATPS*, *MBF1*, *GTPB* and *GST*. The suitability evaluation was executed by the geNorm and NormFinder programs. Overall, *EF1*, *UBCE2*, *EIF5A*, *ATPS* and *60SRPL10* were the most stable genes, and *MBF1*, as well as *MFC*, were the most unstable genes by geNorm and NormFinder software in all experimental samples. To verify the validation of RGs selected by the two programs, the expression analysis of 7 *CtFAD2* genes in safflower seeds at different developmental stages under cold stress was executed using different RGs in RT-qPCR experiments for normalization. The results showed similar expression patterns when the most stable RGs selected by geNorm or NormFinder software were used. However, the differences were detected using the most unstable reference genes. The most stable combination of genes selected in this study will help to achieve more accurate and reliable results in a wide variety of samples in safflower.

## Introduction

Safflower (*Carthamus tinctorius* L.) belongs to the Asteraceae family is a multipurpose plant. Safflower is used in traditional Chinese medicine (TCM) [1]. Most of the medicinal effects of the extract come from the water soluble yellow pigment ‘carthamidin’ [2]. Furthermore, safflower is an ancient oilseed crop widely grown for its high-quality esculent oil [3]. Two major fatty acids—oleic acid (C18:1^Δ9^) and linoleic acid (C18:2^Δ9,12^)—are found in safflower seed oil, which together account for approximately 90% of the total fatty acids in this plant. Safflower oil is well known for its relatively high level of linoleic acid content of more than 70% of total fatty acids (FA) [4–7]. With the rapid development of molecular biology techniques, there has been a tendency to focus on the expression patterns of key genes of interest in safflower, such as genes that regulate the synthesis of flavonoid compounds, oleic acid and linoleic acid, for developing new and exceptional cultivars from the perspective of genetic engineering and plant breeding [8–9].

Transcriptome analysis techniques, including RNA-seq and microarrays, can offer a representative snapshot of the transcriptome that can only be applied to the limited treatments or tissues. Real-time PCR has become an important method for in-depth analysis of gene expression due to its wider range of quantification, higher accuracy, and lower cost [10–13]. However, the accuracy of the results of relative quantification of gene expression is affected by many factors, such as primer design, experimental operation, and choice of optimal annealing temperature, but the choice of optimal RGs has become the most important factor [14]. An erroneous quantification result may emerge when using unstable RGs for normalization in gene expression analysis [15].

Ideal RGs should be expressed at a constant level and not be affected by various experimental elements. The theoretical range of the RG expression levels should be similar to the target genes [16–18]. β-actin, glyceraldehyde-3-phosphate hydrogenase (*GAPDH*), ubiquitin (*UBQ*), elongation factor-1α (*EF1-α*) and *18s rRNA* are common RGs that are often used in quantification experiments [19–24]. However, it is reported that there are no universal RGs under variable experimental conditions. Some common RGs are not constantly expressed under different experimental conditions in different tissues, especially in different species [25–27]. Therefore, it is necessary to systematically evaluate the stability of candidate RGs for diverse experimental conditions prior to their use in RT-qPCR normalization [28].

During the past few years, there have been a great number of reports related to the identification and evaluation of appropriate RGs emerged for plants, such as papaya [29], citrus [30], switchgrass [31], strawberry [32], coffee [33], pea [34], peanut [35], tobacco [36], tomato [37], soybean [38] and arabidopsis [39]. However, so far, there are no reports on the identification and evaluation of RGs in safflower under various experimental conditions. Based on the results of transcriptome analysis in safflower in our research group (unpublished data), some genes with relative stable expression levels at different seed developmental stages were selected as candidate RGs. These genes are the ABC superfamily (*ABCS*), 60s ribosomal protein L10 (*60sRPL10*), ran-binding protein RANBP1 and the related RanBD domain proteins (*RanBP1*), ubiquitin-protein ligase (*UBQL*), multifunctional chaperone (*MFC*), ubiquitin-conjugating enzyme E2 (*UBCE2*), translation initiation factor 5A (*EIF5A*), acetyl-CoA acetyltransferase (*COA*), elongation factor 1 beta/delta chain (*EF1β*), elongation factor 1 alpha (*EF1*), glyceraldehyde 3-phosphate dehydrogenase (*GAPDH*), F0F1-type ATP synthase, beta subunit (*ATPS*), transcription factor MBF1 (*MBF1*), GTP-binding ADP-ribosylation factor Arf1 (*GTPB*), and glutathione *S*-transferase (*GST*). Multiple abiotic stresses, such as gibberellin and paclobutrazol spraying with different concentrations, cold stress, and salt stress, were applied in this study. Three safflower cultivars that have different contents of linoleic acid were used, and various tissues were selected for this study. To evaluate the stability of candidate RGs more appropriately, both geNorm3.5 [40] and NormFinder [41] were used for the analyses. Furthermore, in order to validate the newly identified RGs, a detailed expression analysis of *CtFAD2* genes that control the formation of linoleic acid were performed using both the most stable and unstable reference genes for normalization. Although we could not identify any single gene expressed constantly even in a particular species, one or two appropriate and stable RGs in specific given conditions used in RT-qPCR experiments could be selected. The selection and validation of candidate RGs in RT-qPCR experiments under different experimental conditions should be carried out before they are utilized for the normalization of RT-qPCR data. In this work, the referential recommendations for using these candidate genes are offered to confirm an accurate normalization of transcript level under a particular condition in gene expression studies in safflower by RT-qPCR.

## Materials and Methods

### Plant Materials

Safflower (Carthamus tinctorius L.) accessions PI401477 (81.84/11.13, % oleic/linoleic), PI470942 (76.7/14.3) and PI544021 (16.47/79.1) was provided by Dr. Bradley of American Germplasm Resource Information Network (GRIN). Safflower cultivar Chuanhong No.1 was provided by Ms. Tang Li from Yaan San Jiu Medicine Co. Ltd. Young leaves were taken from 1-month-old seedlings, and for the reproductive organ, seeds were harvested at 0, 10, and 20 days after flowering (DAF). All plant materials were immediately frozen in liquid nitrogen after harvesting and then stored at -80°C until the total RNA was isolated.

### Stress treatments

Samples were collected under various stress conditions. A summary of the stress assays is shown in Table 1. In our previous experiment, safflower seeds were planted in the field in September and were spayed by different concentrations gibberellin acid (GA_3_) and paclobutrazol (PP_333_) at the bud stage in the next year. The results show that 0.09 mM GA_3_ and 0.51 mM PP_333_ can significantly improve the linoleic acid and oleic acid contents of seed oil, respectively, in safflower accession PI544021, so 0.09 mM GA_3_ and 0.51 mM PP_333_ were selected and the seeds at 0 and 10 DAF were used for this experiment.

**Table 1:** Summary of stress assays used to select candidate genes for normalization in safflower.

Biological process	Cultivar	Experimental conditions	Sampled tissue	Biological stages / Time points after treatments
GA_3_ spraying	PI544021	GA_3_ concentration: 0.09 mM	Seeds	0 and 10 DAF after spraying in bud stage
PP_333_ spraying	PI544021	PP_333_ concentration: 0.51 mM	Seeds	0 and 10 DAF after spraying in bud stage
Cold response	PI401477, PI470942 and PI544021	12°C for 48 h	Seeds	10 and 20 DAF after cold stress in bud stage
Salt stress	Chuanhong No.1	NaCl concentration:100mM,150 mM	Leaves	0, 6 and 12 h after stressing in seedling stage
ABA spraying	Chuanhong No.1	ABA concentration: 100 μM	Leaves	0, 2 and 5 h after spraying in seedling stage
Different developmental stages	PI544021	Normal development	Seeds	0, 10 and 20 DAF in bud stage
Different tissues	PI401477	Normal development	Flowers, seeds, leaves	0 DAF in bud stage

**Note:** DAF: days after flowering. GA_3_: gibberellin acid. ABA: abscisic acid. PP_333_: paclobutrazol. The same abbreviations are used in the following tables.

Safflower accessions PI401477, PI470942 and PI544021 were subjected to cold treatments. The plants was grown in the field and at the bud stage were transferred to 12°C for 48 h in an illumination incubator (25°C, 16 h lighting/day). After 48 h, the plants were continued to grow at room temperature, and the seeds were collected at 10 and 20 DAF.

Safflower cultivar Chuanhong No.1 was subjected to a salt stress treatment and abscisic acid (ABA) treatment. For the salt stress treatment, a progressive increase in the salt concentration in the solution (0, 100, 150 mM NaCl) was applied for the 1-month-old seedlings grown in illumination incubator (25°C, 16 h lighting/day). Leaves were taken at 0, 6, and 12 h after reaching the last salt solution concentration. For ABA treatment, 1-month-old seedlings (the growth conditions were the same as above) were sprayed with 100 μM ABA, and the leaves were collected at 0, 2, and 5 h after ABA spraying.

For different seed development stage samples, safflower accession PI544021 was planted under normal conditions, and seeds were selected at 0, 10, and 20 DAF.

Different tissue samples were selected from safflower accession PI544021 under normal development conditions. Seeds and leaves were selected at 10 DAF, and the flowers were selected at 0 DAF.

All experiments were performed with biological triplicates. Details of the above-mentioned experimental conditions are summarized in Table 1.

### Total RNA isolation and cDNA synthesis

A total of 10 mg of plant tissue was used for RNA isolation. Total RNA was extracted using the Trizol method (Trizol was purchased from TaKaRa, Dalian) and then treated with DNAse I for digestion using the RNAse-free kit (TaKaRa, Dalian) to decrease potential DNA contamination. The quality of the RNA samples was verified by 1.0% agarose gel electrophoresis (AGE). The RNA concentration and purity were determined with a NanoDrop ND-2000 spectrophotometer. RNA samples with a 260/230 ratio higher than 2.0 and 260/280 ratio between 1.8 and 2.1, as well as 18S and 28S ribosomal RNA bands with a concentration ratio of approximately 1:2, were selected and used in the subsequent experiment. First-strand cDNA was synthesized using the PrimeScript RT reagent Kit with gDNA Eraser (TaKaRa, Dalian) according to the manufacturer’s instructions. The final cDNA products were diluted for RT-qPCR experiments.

### Selection of Potential RGs and Primer Design in Safflower

On the basis of the results of transcriptome sequencing in safflower with the different seed development stages in our research group (data unpublished), combined with commonly used RGs, some of the stably expressed genes according to the transcriptome analysis results were selected as candidate RGs to seek better RGs with wider adaptation in various experimental conditions.

**Fig 1:**
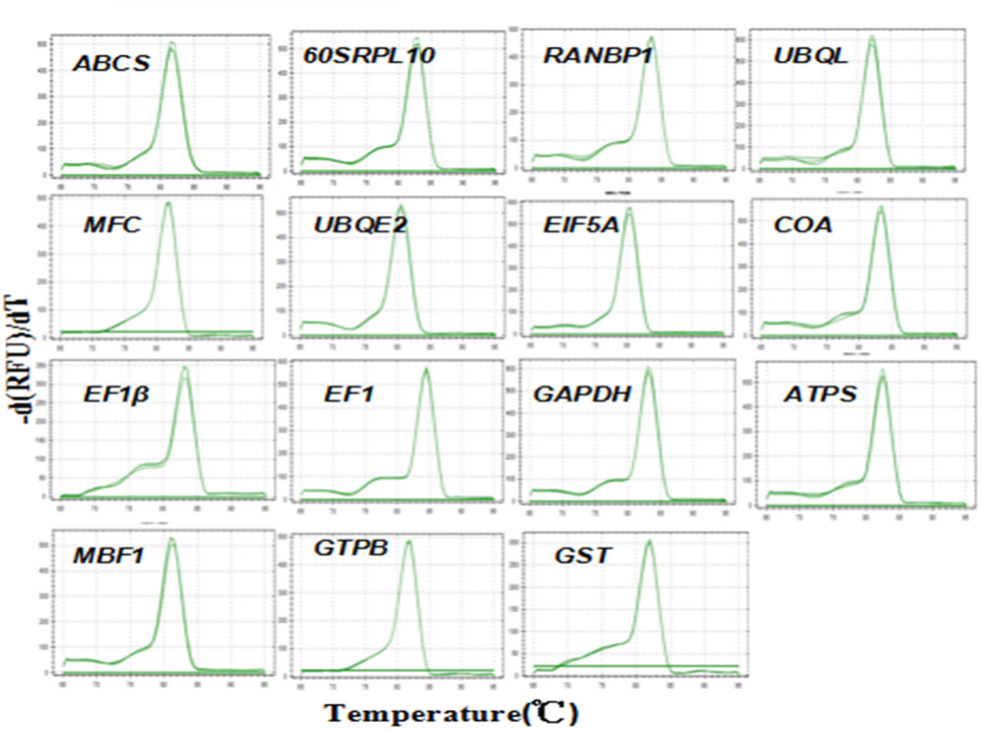
The melting curves of the 15 candidate RGs. A single peak is shown (each including 3 technical replicates of the cDNA pool of the total samples used in this study).

**Fig 2:**
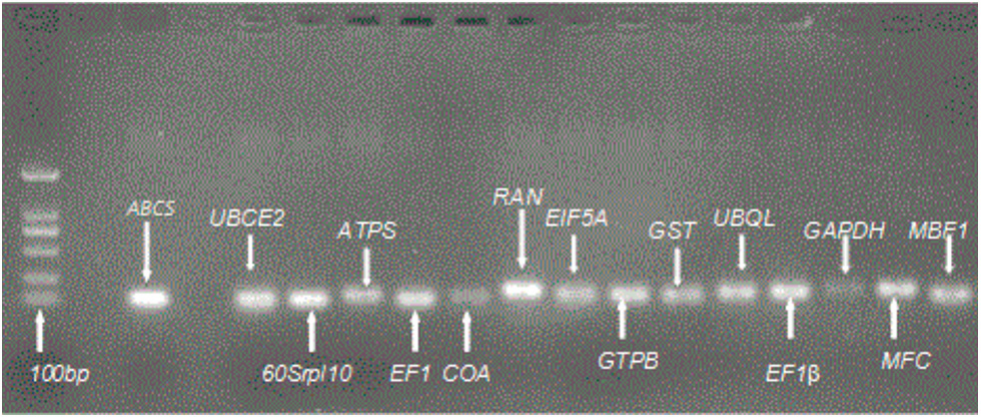
The results of agarose gel (1.0%) electrophoresis of RGs. The amplification of a single product of the expected size for each gene tested in this study is shown.

Primer 3 (v.0.4.0) (http://bioifo.ut.ee/primer3-0.4.0/) online version was used for specific primer design for RT-qPCR using the default parameters for a qPCR experiment. The primers were selected with no hairpins or dimer, and target specificity was detected by Blastn against the nt/nr databank. The primer lengths were between 20–22 bp, the amplicon lengths varied from 90 to 150 bp, and the melt temperatures were between 58 and 63°C. The names, transcriptome numbers, primer and amplicon lengths, melt temperature and primer sequences of the fifteen candidate RGs are shown in Table 2 and Table 3. All primer amplification efficiencies were between 90 and 110% from real-time PCR results. A single peak in the melting curve of the amplification products of each candidate gene and a single band of AGE verified indicates that a single PCR product was amplified effectively and that the specificity of each primer was good. The single peak in the melting curves and the result of the agarose gel electrophoretogram are shown in Figs 1 and 2.

**Table 2:** The primer description of 10 candidate RGs evaluated in this study.

unigene number	gene symbol	gene description	sequence of forward (F) and reverse (R) primers	Amplicon length(bp)	Tm of primer	GC%
comp41339_c0	*ABCS*	Predicted transporter (ABC superfamily)	F 5'-CTTTGGCACGTGCTCTGTTC-3'	101	61.97	55
			R 5'-AAGGGTTTCTTCCAGCCACA-3'		61.94	50
comp35046_c0	*60sRPL10*	60s ribosomal protein L10	F 5'-GTTAGCATCGGTCAAGTCCTTC-3'	98	60.14	50
			R 5'-CCAGGGAACTTGAACTTAGCAC-3'		60.16	50
comp23736_c0	*RanBP1*	Ran-binding protein RANBP1 and related RanBD domain proteins	F 5'-TGCTTCCAGAACCTCCGATT-3'	140	62.05	50
			R 5'-GCTCCGGTGTCTTCGTCTTC-3'		62.3	60
comp36845_c0	*UBQL*	Ubiquitin-protein ligase	F 5'-ACCACCAGCTCCAATCACCA-3'	137	61.43	55
			R 5'-CGCTGCAAGAGGTAGGAGGA-3'		61.32	60
comp38667_c0	*MFC*	Multifunctional chaperone (14-3-3 family)	F 5'-ACATGGCCAAGCTCTCCGAG-3'	130	62.25	60
			R 5'-TAGGCGACGGAGAGGAGGTT-3'		62.21	60
comp31883_c1	*UBCE2*	Ubiquitin-conjugating enzyme E2	F 5'-GAGATGGCACCGTGAGTTATG-3'	102	60.53	52.38
			R 5'-GCCCTTCATGTACAGAGTTGTG-3'		59.66	50
comp40996_c0	*eIF-5A*	Translation initiation factor 5A (eIF-5A)	F 5'-TGTCCCTCATGTCAACCGTA-3'	120	59.96	50
			R 5'-GCATCATCAGTTGGGAGCTT-3'		60.23	50
comp33305_c0	*COA*	Acetyl-CoA acetyltransferase	F 5'-AACGGGGTTGCAAGTCCTGA-3'	118	61.35	55
			R 5'-CAAGTGGCGGTTGGAATGGG-3'		60.89	60
comp18801_c0	*EF1β*	Elongation factor 1 beta/delta chain	F 5'-TCTGGTGTCACTGCTGAAGG-3'	146	60.02	55
			R 5'-TCCTCACCGAAAAGATCCAC-3'		60.05	50
comp40939_c0	*EF1*	elongation factor 1 alpha	F 5'-GTGGTGGGCATCCATCTTGTT-3'	134	62.1	52.38
			R 5'-TACCTCCCAGGCTGATTGTG-3'		62.38	55

**Table 3:** The primer description of 5 candidate RGs evaluated in this study.

unigene number	gene symbol	gene description	sequence of forward (F) and reverse (R) primers	Amplicon length(bp)	Tm of primer	GC%
comp40978_c0	*GAPDH*	Glyceraldehyde 3-phosphate dehydrogenase	F 5'-CTGCCTTGCCCCTCTTGCTA-3'	141	62.2	60
			R 5'-GCAGCTCTTCCACCTCTCCA-3'		61.55	60
comp31267_c0	*ATPS*	F0F1-type ATP synthase, beta subunit	F 5'-CCTGCTGACGATTTGACAGA-3'	147	59.98	50
			R 5'-ATGGGGTGAGAGCATACGAG-3'		60.1	55
comp41245_c0	*MBF1*	Transcription factor MBF1	F 5'-CTATCATGCAGGGCCGTACT-3'	104	60.12	55
			R 5'-AGCCTTCCCGGATTCATACT-3'		59.92	50
comp40111_c0	*GTPB*	GTP-binding ADP-ribosylation factor Arf1	F 5'-GGGTCTCGATGCAGCTGGTA-3'	142	61.68	60
			R 5'-ACCACCAACATCCCACACAGT-3'		61.88	52.38
comp31791_c0	*GST*	Glutathione S-transferase	F 5'-TTTCCGTGGCCCAGAGATCC-3'	98	61.91	60
			R 5'-TCTGTGCTTCATCCGAGAGA-3'		58.16	50

### RT-qPCR analysis

RT-qPCR analysis was performed using the Bio-Rad CFX96 real-time system. Each PCR reaction contained 5 μl SsoFast EvaGreen supermix (Bio-Rad), 1 μl of the diluted cDNA reaction mixture (The cDNA diluted to 200 ng/μl was used for the qPCR assay), 1 μl specific primer with a concentration of 200 nM and 3 μl ddH_2_O in a total reaction volume of 10 μl. Reactions were performed at 95°C for 1 min, 40 cycles of 95°C for 10 s, and 58°C for 30 s in 96-well reaction plates. The specificity of PCR reactions was verified by a melting curve analysis of each amplified sample product. Each real-time PCR reaction was performed in triplicate.

### Stability analysis of reference gene expression

The fluorescence raw data were produced and preserved by Bio-Rad CFX96 Manager 3.0 software. The rapid calculation of threshold cycles and amplification efficiency for every gene was performed. Only genes with amplification efficiency between 90 and 110%, a single peak in their melting curves and a single band in the agarose gel electrophoretogram were selected for the following further analysis.

Because no RGs were used in this experiment, relative quantitative data were acquired by the delta-Cq method for the following geNorm and NormFinder analysis. Delta-Cq data were calculated in Microsoft Excel 2010 using the lowest Cq value (the highest expression value) as the calibrator. The relative quantities were imported to the gene expression stability analysis program geNorm3.5 [40] and NormFinder [41].

The geNorm VBA applet for Microsoft Excel can calculate a gene expression normalization factor (NF) for each sample on the basis of the geometric mean of the minimal number of RGs, estimates of an expression stability value (M) for a reference gene as the average pairwise for that a particular gene with all other tested RGs, and an estimate of the pairwise variation (V-value), Vn/Vn+1, reflecting the effect of additional new genes on the normalization factor. The lower the M value is, the greater the stability of the RG set. Stepwise removal of the gene with the lowest expression stability (the highest M value) allows ranking of the tested genes based on their expression stability. Based on the principle of the lowest number of genes, Vn/Vn+1 with a pairwise variation (V value) lower than 0.15 was accepted as the optimum number of RGs [42–43].

NormFinder software was also used to identify the optimal normalization gene among a set of candidate genes. NormFinder uses a solid statistical framework to estimate both inter- and intra-variations in a given sample set or given experimental design. The best combination of two RGs was given, the stability value for each gene was provided, and the systematic error introduced by using the gene for normalization were evaluated by NormFinder [44]. In most cases, the use of two RGs was more accurate than only one most stable gene. Those candidate genes with the lowest variation both within and between the groups were considered the most stable RGs.

### Determination of the CtFAD2 expression profile

In order to validate whether the most stable genes selected by the geNorm and NormFinder programs were the most suitable for quantification normalization, different RGs were used for normalization in the *CtFAD2* expression profile. The enzyme encoded by *CtFAD2* is primarily responsible for the synthesis of linoleic acid from oleic acid in seed storage lipids. An usually large *CtFAD2* gene family with 11 members from safflower has been isolated according to a previous report [45]. Eight of these genes, *CtFAD2-1*, *CtFAD2-2*, *CtFAD2-3*, *CtFAD2-4*, *CtFAD2-5*, *CtFAD2-6*, *CtFAD2-7* and *CtFAD2-8*, have been cloned in our research group by Ling-Liang Guan [46]. Seven of the genes were selected for expression analysis in this study. The primer information and accession numbers of these genes in NCBI are shown in Table 4. For comparing the differences of the expression profile of seven *CtFAD2* genes, the seeds under cold stress and normal temperature conditions were collected at 10 and 20 DAF, respectively. The cold stress was the same as the above description.

**Table 4:** The primer information of *CtFAD2* genes used in this study for validating the most stable RGs.

Primer name	Nucleotide sequence (5′-3′)	gene name	Accession no.
PF21-4	TTCGTCCTCTACTACCTTGCC	Ct*FAD2-1*	HM165274
PR21-4	CGCCGATGACTGTATTTCC	Ct*FAD2-1*	
PF22-4	TTCCACAACATCACCGACAC	Ct*FAD2-2*	HQ179940
PR22-4	TCCTTCACCTCCTCATCTTTATC	Ct*FAD2-2*	
PF23-4	TGGAGTCTTTGGCACTTTGT	Ct*FAD2-3*	HQ179941
PR23-4	CCGTGGAATCGTAGTGAGGG	Ct*FAD2-3*	
PF24-4	TCCAACACTTCACCACCAG	Ct*FAD2-4*	HQ831351
PR24-4	GGAGCCAAACGACCATAT	Ct*FAD2-4*	
PF25-4	TTTGCTCCCTCCCGCCCTCT	Ct*FAD2-5*	HQ831352
PR25-4	AGCCAACGGCATCGTCCACC	Ct*FAD2-5*	
PF26-4	GGGAGCAGGTGGTCGGATGT	Ct*FAD2-6*)	HQ831353
PR26-4	CGCCAGTGGAGTAGGAAGTTGAG	Ct*FAD2-6*	
PF28-4	AAGCCCAACAAACAAACCAT	Ct*FAD2-8*	HQ831355
PR28-4	CACCCTTGAACGATCCAGTAA	Ct*FAD2-8*	

The mean value of quantified gene expression was calculated from three biological and three technical repeats per sample. The normalized *CtFAD2* gene expression data were calculated by dividing the target gene’s raw expression value for each sample by the appropriate normalization factor.

## Results

### Expression profile of the candidate RGs

**Fig 3:**
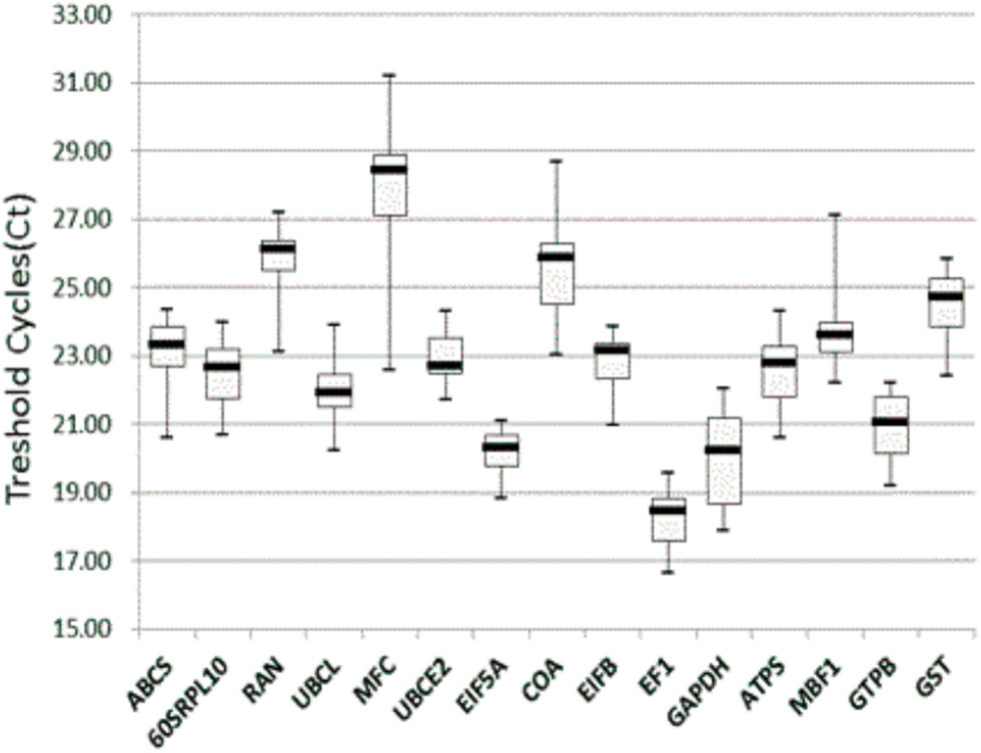
Threshold cycle (Ct) values of the candidate RGs across the experimental samples. A box-plot graph of Ct values shows the median values as lines across the box. Upper and lower boxes indicate the 75th percentile to the 25th percentile. Whiskers represent the maximum and minimum values.

In a qPCR experiment, the cycle at which the fluorescent signal is significantly different from the background was considered the threshold cycle (Ct) value. The Ct value also reflects the difference in the transcription level in some ways. The mean Ct values for the 15 RGs in the 29 samples under study were used to compare the expression rates within genes and the set of samples. As shown in Fig 3, the data exhibited a wide range of expression levels from 16.65 to 31.22 in all samples, and the majority of the selected candidate genes exhibited Ct values ranging from 18.83 to 25.83. Within all samples, the expression variation of a single gene was lower in *EF1*, *EF1β*, *UBCE2*, and *EIF5A* and higher in *MFC*, *COA*, *MBF1* and *GAPDH*. The *MFC* with the variation value of 8.62 and the *EIF5A* with the variation of 2.24 were the highest and lowest variation gene, respectively. The genes with lower expression levels were *MFC*, *RANBP1*, and *COA* with mean Ct values of 27.82, 25.92, and 25.53, respectively. The genes with higher expression levels were *EIF5A*, *GAPDH*, and *EF1* with mean Ct values 20.25, 19.96, and 18.30, respectively. Box-plot analysis only provides an approximate estimate of the stability of candidate RGs, so it is necessary to perform a further analysis in order to identify the most suitable reference gene or the best combination of genes for qPCR experiments under different experimental conditions.

### Stability analysis of expression

**Fig 4:**
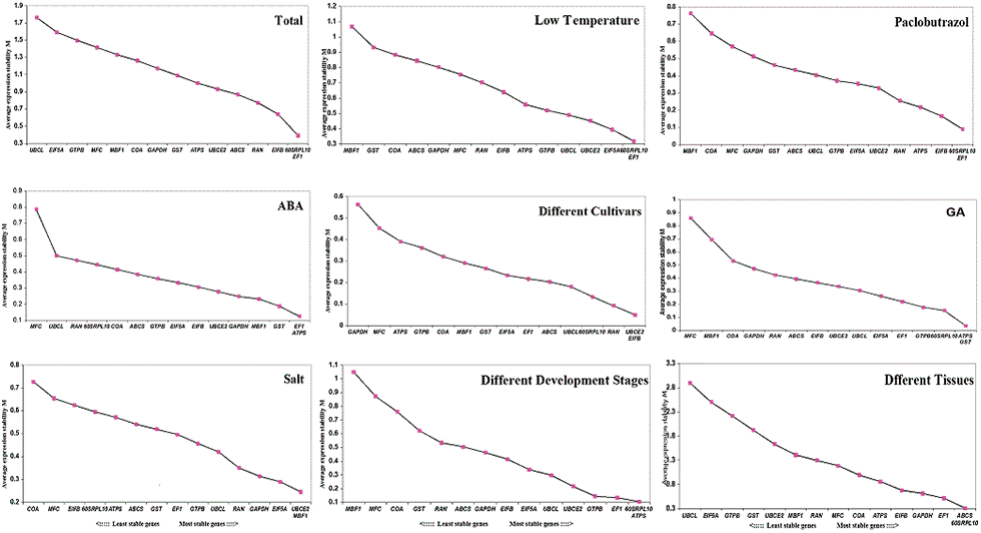
Gene expression stability values (M) of the candidate RGs calculated by geNorm. The figure shows the ranking of the gene expression stability performed in ABA stress, GA_3_ stress, paclobutrazol stress, cold stress, different cultivars, different development stages, and total samples. The most stable genes are on the right, and the least stable genes are on the left. GA_3_: gibberellin acid. ABA: abscisic acid.

geNorm software was used for stability analysis of gene expression under different sets of samples: 1) ABA stress samples, 2) salt stress samples, 3) cold stress samples, 4) GA_3_ stress samples, 5) PP_333_ stress samples, 6) different cultivars, 7) different seed development stages, 8) different tissues, and 9) total samples. Based on the mean expression stability value (M), 15 candidate genes were ranked under different experimental conditions and are shown in Fig 4. High expression stability values with an M value lower than the cut-off (M≤0.15) established by Vandesompele et al [40] for 15 genes are presented. When all of the samples were taken together, the average expression stability value (M) for *60SRPL10* + *EF1* was the lowest, and that of the *MFC* was the highest. This indicated that *60SRPL10* + *EF1* had the most stable expression and *MFC* was the most variably expressed. In addition, *60SRPL10* + *EF1* had the lowest average expression stability value, and *MBF1* had the highest M value both under the cold stress and PP_333_ stress. For salt stress samples, *UBCE2* + *MBF1* present the lowest expression stability value, and *COA* had the highest. When only different cultivates were considered, the *UBCE2 + EF1β* were the most stable genes and *GAPDH* was the most unstable gene in stability analysis. For GA_3_ stress samples, *ATPS* + *GST* was the most stably expressed among the 15 RGs, and *MFC* was the most variably expressed. For ABA stress samples, the most stable combination was *EF1 + ATPS*, and *MFC* was the most unstable gene. For different seed development stage samples, *60SRPL10* + *ATPS* were considered the most stable combination of genes and *MBF1* was the most unstable gene according to the geNorm analysis results. Finally, when different tissues were considered for stability analysis, *ABCS* + *60SRPL10* was the most stable combination of RGs, and *UBCS* was the most unstable gene.

**Fig 5:**
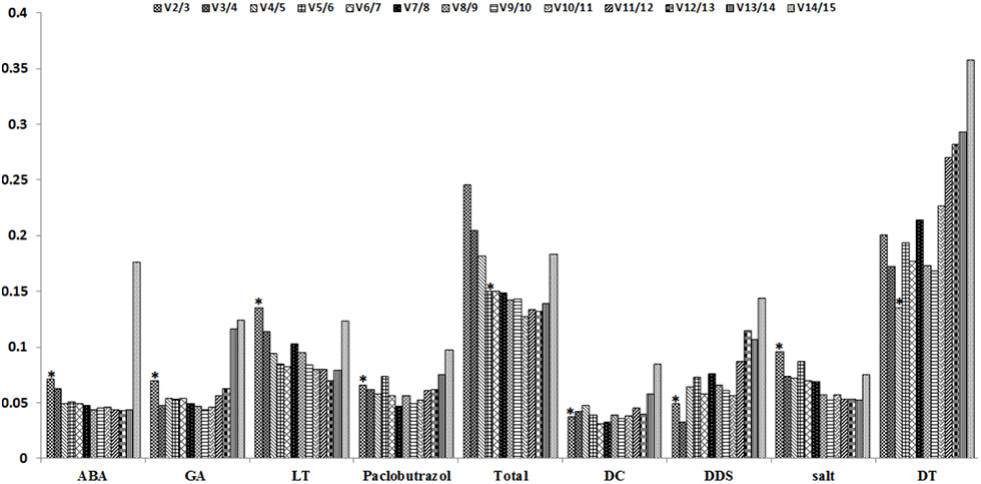
Gene expression pairwise variation (V) of the candidate RGs calculated by geNorm. Pairwise variation (V_n_/V_n+1)_ was analyzed between the normalization factors NF_n_ and NF_n+1_. Asterisk indicates the optimal number of RGs required for normalization. ABA (abscisic acid stress), GA_3_ (gibberellin stress), LT (low temperature), Paclobutrazol (paclobutrazol stress), Total (total samples), DC (different cultivates), DDS (different development stages), Salt (salt stress), DT (different tissues).

The optimal number of RGs was also calculated by geNorm automatic analysis for accurate normalization. Under different experimental conditions, the optimal number of RGs may be different than the results of pairwise variation analysis (Fig 5). Except for the total samples and the different tissue samples, the V_2/3_ values of the other sets of samples were all lower than the cut-off of 0.15, indicating that it is sufficient to use two RGs for accurate normalization. However, when pairwise variation was evaluated in all samples, the V_2/3_ value was 0.158, the V_3/4_ value was 0.205, and the V_4/5_ value was 0.182. The V_5/6_ value was 0.15, so the five most stable RGs, including *60SRPL10*, *EF1*, *UBCE2*, *EIF5A* and *ATPS*, should be used for more accurate normalization. For different tissues samples, the V3/4 value was 0.172 and the V 4/5 value was 0.135, so it was better to use four RGs for normalization.

NormFinder was also used to analyze the expression stability and to rank the stability value from high to low; the ranking of all candidate RGs in different set of samples is shown in Table 5 and Table 6. When all 29 samples sets were considered, *EIF5A* was the most stable gene with a stability value of 0.060, and the best combination of two genes was *EF1 + EIF5A* with a stability value of 0.050. For ABA stress samples, the best gene was *UBCE2* with a stability value of 0.180, and the best combination of two genes was *60SRPL10* + *UBCE2* with a stability value of 0.141. For GA_3_ and PP_333_ stress samples, the best combinations of two genes were *GST + ATPS* and *ATPS + UBCE2* with a stability value of 0.014 and 0.060, respectively. *ATPS* represented the best stability in the previous two sets of samples. For salt stress, the best combination of genes was *EF1 + GTPB* with a stability value of 0.093, and the best gene was *EF1* with a stability value of 0.132. When only low temperature stress samples were considered, NormFinder identified that the most stable expression gene was *EIF5A* with a stability value of 0.079, and the best combination of two genes was *EIF5A + 60SRPL10* with a stability value of 0.082. Finally, because the different cultivars, different seed development stages samples and different tissue samples had no control samples and NormFinder raw data cannot be divided into two groups, NormFinder did not calculate the most stable combination of two genes for all candidate RGs (Table 2 and Table 3). Thus, when different seed development stage samples and different cultivars were considered, *EF1β* + *UBCE2* were the most stable RGs with stability values of 0.025 and 0.053, respectively. For different tissue samples, *MBF1* + *RAN* were the most stable genes and *MFC* was the least stable gene in ABA, GA_3_, and all the sets of samples. Under the different seed development stages, cold and PP_333_ stress samples, *MBF1* had the highest stability value. *COA* + *GAPDH* were the least stable genes under salt stress and in different cultivate samples, respectively. In addition, *UBQL* was considered the most unstable gene for different tissue samples by NormFinder.

**Table 5:** Expression stability values of safflower RGs as calculated by NormFinder software.

Total		ABA		GA_3_		PP_333_		Salt	
Ranking	Stability value	Ranking	Stability value	Ranking	Stability value	Ranking	Stability value	Ranking	Stability value
*MFC*	0.431	*MFC*	3.392	*MFC*	1.251	*MBF1*	0.769	*COA*	0.932
*MBF1*	0.381	*ABCS*	0.911	*MBF1*	1.247	*COA*	0.742	*ABCS*	0.444
*COA*	0.377	*EF1β*	0.827	*COA*	0.713	*GAPDH*	0.581	*GAPDH*	0.367
*GAPDH*	0.374	*ATPS*	0.696	*GAPDH*	0.690	*MFC*	0.412	*UBQL*	0.299
*GST*	0.210	*EF1*	0.669	*RAN*	0.650	*UBQL*	0.339	*ATPS*	0.284
*ABCS*	0.209	*UBQL*	0.666	*ABCS*	0.583	*GST*	0.307	*60SRPL10*	0.251
*ATPS*	0.193	*COA*	0.652	*EF1β*	0.542	*RAN*	0.258	*UBCE2*	0.238
*UBCL*	0.177	*RAN*	0.649	*UBCE2*	0.521	*ABCS*	0.248	*EF1β*	0.231
*GTPB*	0.164	*EIF5A*	0.567	*UBQL*	0.504	*EF1β*	0.222	*GST*	0.210
*RAN*	0.153	*GAPDH*	0.430	*EIF5A*	0.411	*60SRPL10*	0.163	*MFC*	0.206
*EIFB*	0.150	*GTPB*	0.389	*EF1*	0.286	*EIF5A*	0.163	*RAN*	0.195
*60SRPL10*	0.121	*MBF1*	0.275	*GTPB*	0.150	*GTPB*	0.159	*EIF5A*	0.184
*UBCE2*	0.110	*GST*	0.260	*60SRPL10*	0.077	*EF1*	0.129	*MBF1*	0.161
*EF1*	0.081	*60SRPL10*	0.202	*GST*	0.031	*UBCE2*	0.110	*GTPB*	0.132
*EIF5A*	0.060	*UBCE2*	0.180	*ATPS*	0.009	*ATPS*	0.031	*EF1*	0.132
**Best gene**	**Stability value**	**Best gene**	**Stability value**	**Best gene**	**Stability value**	**Best gene**	**Stability value**	**Best gene**	**Stability value**
*EIF5A*	0.060	*UBCE2*	0.180	*ATPS*	0.009	*ATPS*	0.031	*EF1*	0.132
**Best combination**	**Stability value**	**Best combination**	**Stability value**	**Best combination**	**Stability value**	**Best combination**	**Stability value**	**Best combination**	**Stability value**
*EIF5A* and EF1	0.050	*60SRP10* and *UBCE2*	0.141	*ATPS* and *GST*	0.014	*ATPS* and *UBCE2*	0.060	*EF1* and *GTPB*	0.093

**Table 6:** Expression stability values of safflower RGs as calculated by NormFinder software.

Cold		DDS		DFC		DT	
Ranking	Stability value	Ranking	Stability value	Ranking	Stability value	Ranking	Stability value
*MBF1*	0.729	*MBF1*	2.147	*GAPDH*	1.264	*UBQL*	5.338
*GST*	0.433	*MFC*	1.617	*MFC*	0.883	*EIF5A*	3.655
*MFC*	0.378	*COA*	1.465	*COA*	0.515	*UBCE2*	3.404
*ATPS*	0.372	*GST*	1.093	*ATPS*	0.461	*GTPB*	2.867
*GAPDH*	0.358	*RAN*	0.606	*GTPB*	0.434	*GST*	2.622
*COA*	0.350	*UBCE2*	0.545	*GST*	0.381	*ABCS*	2.097
*ABCS*	0.332	*GAPDH*	0.481	*MBF1*	0.363	*EF1*	1.926
*GTPB*	0.263	*ABCS*	0.463	*ABCS*	0.268	*60SRPL10*	1.816
*RAN*	0.247	*UBQL*	0.321	*UBQL*	0.230	*GAPDH*	1.614
*UBCE2*	0.242	*EIFB*	0.301	*EF1*	0.221	*COA*	1.377
*UBCL*	0.233	*EF1*	0.276	*EIF5A*	0.131	*MFC*	1.097
*EF1β*	0.201	*GTPB*	0.235	*60SRPL10*	0.071	*EIFB*	1.04
*60SRPL10*	0.144	*EIF5A*	0.179	*RAN*	0.054	*ATPS*	0.467
*EF1*	0.142	*ATPS*	0.069	*UBCE2*	0.025	*RAN*	0.398
*EIF5A*	0.079	*60SRPL10*	0.053	*EF1β*	0.025	*MBF1*	0.398
**Best gene**	Stability value	**Best gene**	Stability value	**Best gene**	Stability value	**Best gene**	Stability value
**EIF5A**	0.079	**60SRPL10**	0.053	**UBCE2/EF1β**	0.025	**RAN/MBF1**	0.398

**Note:** DDS: different development stage, DFC: different cultivars, and DT: different tissues.

### RG validation by quantification of the CtFAD2 expression profile with different normalization factors

In order to validate whether the most stable genes selected by geNorm and NormFinder programs were the most suitable for quantification normalization, different RGs were used for normalization.

*CtFAD2* is the key gene that controls the formation of linoleic acid. Seven *CtFAD2* genes selected in this study had been identified, and their sequence had been submitted in NCBI for safflower. Under cold stress, the content of accumulated polyunsaturated fatty acids (PUFA) in plant tissues will usually increase significantly [47–48].

**Fig 6:**
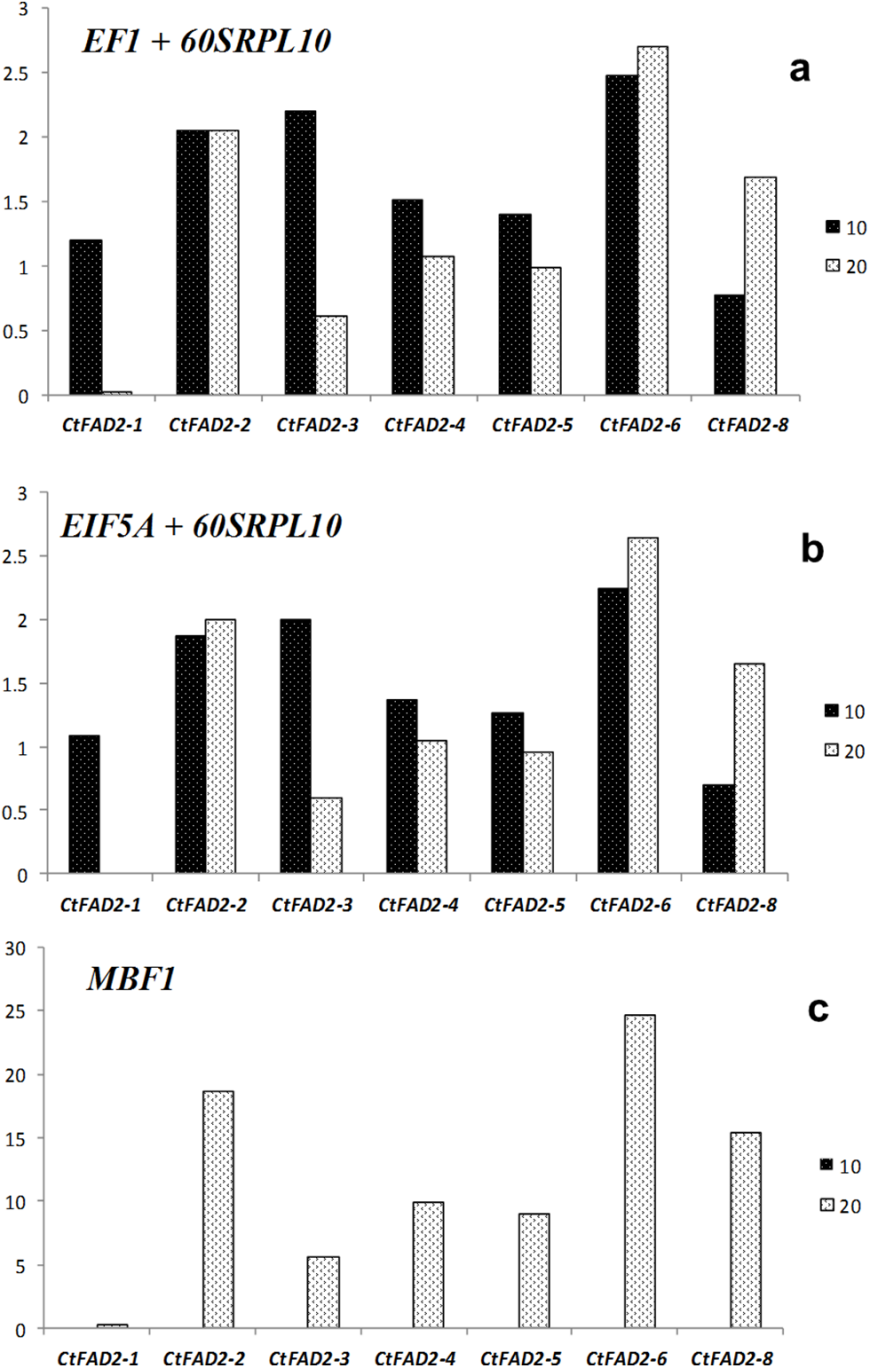
Relative quantification of 7 *CtFAD2* genes using different combinations of RGs or single RGs for normalization. **a.** Gene expression normalized with the most stable RGs selected by geNorm for the low temperature stress samples. **b.** Gene expression normalized with the most stable RGs selected by NormFinder for low temperature stress samples. **c.** Gene expression normalized with the most unstable genes selected by both geNorm and NormFinder for low temperature stress samples.

*60SRPL10 + EF1* was selected as the most stable combination of genes by geNorm algorithms, and *60SRPL10 + EIF5A* were identified as the most stable genes by NormFinder and were used in expression analysis of 7 *CtFAD2* genes under cold stress for normalization (Fig 6A and 6B). *MBF1*, as the least stable gene identified by both geNorm and NormFinder, was also used for normalization to further verify whether the use of unstable RGs can lead to an inaccurate result for quantification expression analysis (Fig 6C). The results showed that the fold changes of *CtFAD2-1*, *CtFAD2-2*, *CtFAD2-3*, *CtFAD2-4*, *CtFAD2-5*, and *CtFAD2-6* were all greater than 1 at 10 DAF, indicating that the expression of these genes under cold stress in safflower seeds was higher than in the control (seeds under normal temperature). These results were in accordance with the result that the relative percentage content of linoleic acid in safflower seed had an obvious increase at 10 DAF under cold stress according to fatty acid analysis by GC-MS. Fig 6C shows that, under cold stress, the expression of all genes was nearly zero at 10 DAF and that the fold change of all genes except for *CtFAD2-1* was extremely high compared with the control at 20 DAF. Overall, it was nearly similar to the expression pattern of 7 *CtFAD2* gene under cold stress at 10 and 20 DAF regardless of whether *60SRPL10 + EF1* or *60SRPL10 + EIF5A* was used for normalization (Fig 6A and 6B). However, when *MBF1* was used as the reference gene for normalization, the result was obviously different from those mentioned previously. Unstable RGs can lead to the NF value becoming larger or smaller, corresponding to the relative expression value of target genes becoming smaller or larger, respectively. Therefore, it is very necessary to use an appropriate reference gene for relative gene expression quantification.

## Discussion

With the development of molecular biology techniques, such as the transcriptome, microarray, digital gene expression profile and real-time quantitative PCR, investigation of gene expression patterns at the transcript level has become feasible. RT-qPCR has become a frequently used technology for specific gene expression analysis [47].

Although *EF1α* and *UBQ* were used as reference genes in safflower gene expression analysis with the RT-qPCR technique, the validation of a suitable set of RGs is still of crucial importance [48–50]. This safflower transcriptome analysis identified a group of stably expressed genes under different seed development stages, and these data resources made it convenient for us to select some relatively stable candidate RGs for normalization in order to further identify and evaluate the most appropriate RGs for stable expression across a variety of experimental conditions. This work is the first particular study aimed at the identification and validation of a series of candidate reference genes for normalization of transcription in safflower. For this study, some candidate RGs that had a relatively more stable and higher expression level based on the transcriptome data were selected in safflower. After determining the AGE and solubility curves, 15 candidate RGs were selected in order to validate their normalization potential among 29 samples under different abiotic stresses and at different developmental stages.

In the present study, geNorm and NormFinder were used to select the best RGs for the normalization of gene expression data in safflower. It is acceptable that there were differences between analysis results regarding the ranking of the candidate RGs from the two programs because of their differences in mathematical approaches. Although the differences in the ranking of genes existed, the most and the least stable genes were nearly consistent under different sets of samples from the two distinct program analysis results. *MFC* was always the least stable gene, whether in ABA, GA_3_-sprayed samples or total samples from the two analysis methods. In addition, *MBF1* was the most unstable gene in cold stress, PP_333_ stress and different seed development stages samples. Although *GAPDH* was commonly used as the RG for normalization in gene expression analysis, in our study, it was the least stable gene in different varieties of safflower, and *COA* was the least stable gene in salt stress samples. This result indicated that the common RGs for normalization should be used carefully before an evaluation for every set of samples under different experimental conditions. From a single gene perspective, combined with the results of two methods, *EF1*, *EIF5A*, *ATPS*, *UBQE2* and *60SRPL10* were strongly recommended as RGs for relative quantification of gene expression in safflower. In addition, *EF1* had been selected as the best reference gene in switchgrass [31], strawberry [32], perennial ryegrass [51], rice [52] *lolium* [53] and *Brachiaria* [54]. However, there were some differences in the best combination of genes for normalization from the analysis results of the two programs. *60SRPl10* + *EF1* was considered the best combination of candidate genes for normalization under total samples, paclobutrazol stress samples, and cold stress samples by geNorm analysis. However, in the results with NormFinder, *EF1 + EIF5A* was identified as the best combination among total samples, *EIF5A + 60SRPL10* was identified as the best combination in cold stress samples and *ATPS + UBCE2* was identified as the best combination in paclobutrazol stress samples. Under ABA stress, *EF1 + ATPS* was identified as the best combination by geNorm and *60SRPL10 + UBCE2* was identified as the best combination by NormFinder. *UBCE2 + MBF1* was the best combination by geNorm, and *EF1 + GTPB* was identified as the best combination by NormFinder under salt stress. Finally, *ATPS* + *GST* was identified as the best combination of genes for normalization by both geNorm and NormFinder programs.

To verify the effectiveness of selected RGs, the seven copies of the *CtFAD2* gene as target gene was used to evaluate RGs efficiency for normalization in cold stress samples. Similar expression patterns appeared when two stable combinations of candidate genes analyzed were used for normalization by geNorm and NormFinder. However, when the least stable gene was used for normalization, the result was obviously different from the previous result. At 10 and 20 DAF, the expression of *CtFAD2* in safflower seeds was lower and highly significant, respectively. The standardized expression quantity of the target gene was acquired by dividing by the normalization factor (NF) of reference gene, so the lower or higher NF can directly lead to an exponential increase or decrease in the expression level. Therefore, the identification and stability evaluation of RGs should be made prior to their application in transcription normalization studies of gene expression. Our study may become a reference for high-efficiency identification and evaluation of RGs based on transcriptome data for other researchers and with other species. Meanwhile, these results also provide a convenient reference for investigations of the expression patterns of key genes of interest in safflower.
